# The relationship between benevolent leadership and affective commitment from an employee perspective

**DOI:** 10.1371/journal.pone.0264142

**Published:** 2022-03-09

**Authors:** Dorota Grego-Planer

**Affiliations:** Department of Enterprise Management, Faculty of Economic Sciences and Management Nicolaus Copernicus University in Torun, Torun, Poland; Universiti Pertahanan Nasional Malaysia, MALAYSIA

## Abstract

Benevolent leadership has emerged as a contemporary leadership style that has been studied only scantly. To fill this gap, this work has two goals. The first is the identification and assessment of the relationship between benevolent leadership and employees’ affective commitment in the context of Polish organizations. Secondly, it will be investigated whether all constructs of benevolent leadership contribute to affective commitment. Data were obtained from 415 company employees. The relationships were investigated using structural equation models (SEMs). Analyses of the results showed that benevolent leadership has a positive relationship with affective commitment. The more benevolent leadership qualities a supervisor has, the more commitment employees show. All dimensions of benevolent leadership are positively correlated with affective commitment. However, the greatest was found in the “community dimension.” All analyzed dimensions correlate positively with each other, so there is a high probability that if a leader displays one BL dimension, he will also display another.

## Introduction

The subject literature pays much attention to different leadership styles. Research on leadership styles has particularly focused on transformational leadership [1, 2], ethical leadership [3], spiritual leadership [4], servant leadership [5] or authentic leadership [6, 7].

While each of these leadership theories emphasizes the importance of morality at its root, they differ quite significantly from each other. Transformational leadership, defined as “the process of influencing major changes in attitudes and assumptions of organizational members and building commitment for the organizations mission and objectives” focuses on a leader–follower relationship that benefits both the individuals involved and the organization as a whole [8]. Authentic leadership is a synergistic combination of self-awareness, sensitivity to the needs of others, creativity, honesty and transparency in relation to oneself and others [9]. Ethical leadership is defined as “The demonstration of normatively appropriate conduct through personal actions and interpersonal relationships, and the promotion of such conduct to followers through two-way communication, reinforcement, and decision-making” [10]. Servant leadership, in turn, is oriented towards others, manifested in the fact that the needs and interests of the followers are given priority, and the concern is directed at others in the organization and in the community [11]. Spiritual leadership is defined as the values, attitudes and behaviors that are necessary to intrinsically motivate oneself and others so that they have a sense of spiritual survival through calling and membership” [12].

All the above-mentioned theories of leadership have played a significant role in the development of a moral approach to managing people and organizations. However, none of them has become dominant and none provides an answer to how to meet the challenges of the current economy. Leadership reorganization must focus on balancing the economy, quality of working life and social responsibility. So far, many researchers have raised topics related to morality, spirituality, positive change or the social responsibility of leaders. However, they have focused their attention only on one area. They have adopted many theories from other disciplines, such as business ethics, spirituality at work, appreciative inquiry, positive organizational scholarship and corporate social responsibility. Each of these concepts touches on the topic of the influence that leaders have on the world around them and tries to help them better cope with the ethical, social and emotional challenges of the economy. Each of these areas also relates to the benevolence of leaders in terms of their authenticity, the promotion of the common good, and integrity. It should be noted, however, that despite the fact that all the above-mentioned theories of leadership and research trends addressed the issues of positive changes in organizations, none of them fully explained the attitudes and behaviors of leaders in terms of their benevolence and desire to contribute to the surrounding world.

Karakas, however, proposed a synthetic approach to all mentioned leadership styles under the term “benevolent leadership” (BL) [13]. This concept treats leadership as a process of initiating positive changes in organizations. Benevolent leaders create visible effects for the common good. They combine morality, spirituality, vitality and community [14].

Karakas defined benevolent leadership as the process of creating a virtuous cycle of encouraging, initiating and implementing positive change in organizations through: a) ethical decision making and moral actions, b) developing spiritual awareness and creating a sense of meaning, c) inspiring hope and fostering courage for positive action, and d) leaving a legacy and positive impact for the larger community [13]. The benevolent leadership model thus differs conceptually from other values-based leadership in that it clearly focuses on creating positive change, especially in human values. It balances the ethical, transformational and social concerns of leaders and provides guidance for leaders to build understanding, “human” organizations. Benevolent leadership shares ethical sensitivity, integrity and self-awareness with ethical leadership, and positive engagement with authentic leadership. It shares spiritual depth, integrity, self-awareness and hope with spiritual leadership. Additionally, benevolent leadership includes community responsiveness, stewardship and wisdom that are in common with servant leadership [15]. As Ghosh claims, “the additional focus on community and social responsibility makes the benevolent leadership a worth exploring approach” [16].

Attention is focused on creating benefits, activities and results for the common good. The emphasis on the common good is critical here, as the very essence of benevolent leadership is focused precisely on creating positive change and engaging in activities that benefit the wider community. The concept of benevolent leadership emphasizes the individual actions and behavior of the leader. It refers to his individual approach to ethics, spirituality, introducing changes and social responsibility.

It should be emphasized, however, that some of the research presented in the literature concerning the BL concept has been perceived differently and originated in Eastern countries. Eastern scholars (mainly Chinese) regard benevolent leadership as a style that conforms to the teachings of Confucius. In the Chinese context, this style means that leaders display holistic caring behavior concerning the personal well-being of their subordinates. According to Farh et al. [17], “benevolent leadership can be demonstrated as a form of individualized care within a work domain, such as allowing opportunities to correct mistakes, avoiding the public embarrassment of subordinates, providing coaching and mentoring, and showing concern for subordinates’ career development.” It can also be expressed as a form of individualized care within a non-work domain, such as treating subordinates as family members, assisting subordinates during their personal crises and showing holistic concern beyond professional relationships [17].

Wang and Cheng emphasize that benevolent leaders’ cultural awareness about deeply entrenched Confucian teachings allows for the practice of mutual obligations in social relations [18]. The personalized care shown by benevolent leaders includes offering opportunities to correct mistakes, avoiding public embarrassment of employees, providing coaching and mentoring, and treating employees as family members. In this approach, they conducted research: for example Cheng et al. [19] or Tan, Zawawi and Aiz [20]. As can be seen from the above description, the differences in these two approaches to benevolent leadership are very significant. According to the Eastern concept, BL means direct care for the employee, while according to the Karakas concept, BL means “caring” for all stakeholders. (The concept of BL proposed by Karakas is adapted to the cultural conditions of Western countries.)

Considering the key role of benevolent leaders in creating the common good, it can be assumed that benevolent leadership may result in positive attitudes and behavior among employees. G. Erben and A. Güneşer stated that the benevolent behavior of leaders encourages commitment. Beneficial actions of superiors inspire employees and also create an emotional bond between the two parties. This emotional relationship discourages employees from leaving their organization [21].

Thus, the author focused her attention on the search for a relationship between benevolent leadership and the affective commitment of employees, by which we mean the sense of emotional attachment to the organization. The literature review clearly shows that this dimension of employee commitment is most relevant to the organization. Many scientists have proved that affective commitment influences the success of an organization. A high level of affective commitment is associated with high employee productivity and low absenteeism [22, 23]. In addition, this dimension of commitment has the greatest impact on organizational citizenship behavior [24]. Highly attached employees are achievement- and innovation-driven, with the ultimate goal of improving performance [25]. Due to the numerous effects of affective commitment of organizations, the knowledge of managers on this subject should become crucial. Leaders should take actions that will trigger this desired attitude in their subordinates. The influence of leadership on affective commitment has been investigated many times. Some scientists have studied the overall effect of leadership on affective commitment in public or private organizations [26, 27]. Others have focused on narrow aspects of leadership style, evidencing that, for example, transactional and transformational leadership are positively correlated with AC [28]. It is therefore worth examining these relationships in benevolent organizations also.

The research presented in the literature, in line with the Karakas concept, shows a research approach focused on the self-assessment of leaders. In line with this approach, the leaders themselves evaluate their benevolence [14, 16]. The author of the article, however, proposed a different perspective for research on this topic, in which a significant assessment of the benevolence of the leader can be obtained from the employees themselves. This approach to the evaluation of leaders is used by many researchers, e.g. Mostafa; Zhang X, Yao Id Z; and Engelbrecht, Heine, Mahembe [29–31]. Jaramillo, Carrillat and Locander argue that when individuals evaluate themselves or their performance they are likely to rely on inner thoughts, feelings and personal attributes [32]. This is why I believe that employees are the most able to indicate how benevolent their superior is. It should be noted that the author consulted Fahri Karakas, the creator of the concept, about the proposal of such an approach to the BL research, and received the reply decided that such a perspective is an extremely interesting direction in the research on benevolent leadership.

To sum up the research presented in this article: first, it concerns only benevolent leadership as a process of initiating positive change (in accordance with the concept of Karakas). Second, it represents a new approach to BL research that includes the employees’ perspective and their evaluation of the benevolence of the leader.

The research presented in this article thus has two goals. The first is the identification and assessment of the relationship between benevolent leadership and affective commitment in the context of Polish organizations. Secondly, it will be investigated whether all dimensions of benevolent leadership contribute to affective commitment. However, these dependencies will be identified from the employees’ perspective. In summary, it should be noted that the study presented in the article is only the first part of a broader project. Further results will be presented in subsequent articles.

## Literature review

### Affective commitment as a special dimension of organizational commitment

Organizational commitment is currently a key research subjects in the area of organizational behavior. This interest among researchers may be due, for example, to commitment being a special type of employee attitude that differs entirely from motivation or job satisfaction. It results from a willing and genuine desire to participate in the organization and to identify with it. In this sense, commitment can, more than other attitudes, influence the effectiveness of actions taken, cooperation or loyalty.

The American sociologist Becker is considered to be the father of research on organizational commitment. However, he treated this concept as an effect of the relationship of economic exchange between employee and organization [33]. The author associated commitment with the expectation of receiving future benefits or maintaining existing ones, having in mind, for example, rewards or special rights. The aforementioned emotional aspect of commitment was only later taken into account in research, including in the work of Mowday, Steers and Porter. These authors defined organizational commitment (OC) as the relative strength of an individual’s identification with and involvement in a particular organization” [34]. Organizational commitment is the employee’s individual attachment to the company and his identification with the environment and values of the organization in which he works. In this sense, organizational commitment is understood as a special kind of bond between organization and employee that is characterized by interdependence and that contributes to the employee’s active participation in the functioning of the company and causes the employee to use his natural abilities and skills to achieve its goals and objectives [35]. O’Reilly and Chatman believe that organizational commitment is the employee’s internal attachment to the organization, as manifested in his attitudes towards the organization [36]. The emotional structure of organizational commitment is also shown in the works of Schaufeli et al. [37]. The author maintains that it is a state that consists in the employee experiencing vigor in performing his work and being dedicated to and absorbed in it.

Researchers dealing with the phenomenon describe it very similarly, focusing primarily on various components and dimensions of organizational commitment.

Porter et al. described the concept of commitment, noting that it occurs when three phenomena occur simultaneously. First, there is strong belief, and the employee’s acceptance of organizational values and goals. Second, the employee is prepared to put significant effort into the organization. Third, there is a strong desire to remain a member of the organization [38]. The works of Kahn [39] were of great importance in elaborating the concept of commitment. In his opinion, commitment is a mental state that enables employees to express themselves during their work. Kahn’s concept was based on the conviction that this self-expression in action can take place in three dimensions: the physical, the cognitive and the emotional. The researcher assumed that each dimension can separately characterize the behavior of an employee and can reach its own level of intensity [39]. However, all dimensions must be present for commitment to occur.

Usually, the phenomenon of organizational commitment is related to employee attitude, which has a psychological basis and is understood as the employee’s strong identification with the organization and the desire to remain part of it. In this context, in the 1990s, Allen and Meyer created a model of organizational commitment that combines three dimensions of this phenomenon. Accordingly, Allen and Meyer theorize, organizational commitment encompasses three dimensions: affective commitment, normative commitment and continuance commitment [40].

Affective commitment refers to the employee’s emotional bond with the organization that causes the employee to identify with the mission, goals, values and principles of the company and to associate his future with it. Affective commitment (AC) is determined by the employee’s personal choice to remain committed to the organization via some emotional identification with the organization. It is a particular and long-lasting commitment for the employee that results from his individual beliefs and recognition of the company. An affective employee takes up employment in a given organization of his own free will [41]. As a result, he has a positive attitude to work and carries out assigned tasks effectively. In this case, belonging to a given organization increases the employee’s self-esteem and makes the work he undertakes a source of fulfillment and satisfaction. Affective commitment increases the employee’s performance [42]. As Wang, Wang Zhou et al. write: “when the employees’ affective commitment level is high, they have a higher degree of identification and affective attachment to the organization, are more willing to contribute to the development of the organization, and focus on making organizational change better so as to make taking change behavior” [43]. It should be added that affective commitment is negatively correlated with absenteeism and employee turnover, as well as with workplace stress [44, 45].

The second dimension of commitment is continuance commitment (CC). This is understood as the employee staying in a given company mainly due to the costs that leaving would incur. In this sense, resigning is too expensive [40]. Faloye points out that the employee who is committed in this dimension assesses only the economic benefits of staying in the organization [46]. Continuance commitment can be regarded as a contractual attachment to the organization [47]. CC develops when the employee sees no alternative employment or when he perceives that he has already invested too much in the organization [48]. The employee with continuance commitment performs his duties less well and has more difficult relationships with his colleagues.

The last dimension of the model is normative commitment (NC). It results from the sense of obligation to remain in the company, and is related to the employee’s loyalty, honesty and solidarity vis-à-vis the company [41]. Here, the employee’s attachment to the organization is connected with the social norms he has adopted and is an expression of his gratitude for and recognition of the company’s prevailing values and principles. The level of NC may be influenced by the rules an individual accepts and the reciprocal relationship between the organization and its employees [49, 50].

The authors of the model assume that the employee’s relationship with the organization can be defined by several dimensions of attachment simultaneously. Strong affective attachment may also be associated with a sense of obligation to remain within the organization. The same employee may also feel that the costs of leaving the job would be excessive [41].

Undoubtedly, however, the type of employee commitment most desirable to organizations is affective commitment, because it is the only one that involves the employee identifying with the company and taking an active part in activities of benefit to it. According to J.R. Pieper, J.M. Greenwald and S.D. Schlachter, affective employees perceive the organization as in line with their own goals and values, which makes them find their workplace attractive and rewarding [51].

Summing up, it should be emphasized that people with a high degree of affective commitment remain in a given organization and bind their future with it because they want to. Moreover, a high level of affective commitment is associated with high employee productivity and creativity and a desire for individual development and collaboration [22, 23]. That is why this dimension of commitment receives such a high level of attention from many researchers. It is also the subject of the author’s research.

### Determinants of affective commitment

The literature emphasizes many theories and research approaches regarding the determinants of the organizational commitment of employees, including affective commitment [52, 53]. They are founded on the assumption that commitment arises when three basic conditions are met–meaningfulness, safety and availability [39]. The first condition, meaningfulness, is understood as the employee’s awareness that his individual work and effort are appropriately recognized. Employees see their actions as having meaning when employers give them to understand that they are necessary and indispensable to the company they are currently in. The second condition, safety, means that the employee desires to be actively involved and is not worried about the consequences for his role or reputation. Meanwhile, the third condition, availability, refers to the employee having specific emotional, mental and physical resources the active involvement of which are required by the company’s operations. The employee’s sense of availability is mainly built on inner strength and vitality, as well as a happy private life.

McBain emphasizes that there are three main factors influencing the creation of organizational commitment in employees–company characteristics, employee attributes, and management that is professional (e.g. has managerial and communication skills) [54].

However, while focusing only on affective commitment, it should be noted that it depends on many factors. One of them is job satisfaction. Affective attachment is also influenced by job involvement. Lodhal and Kejner define commitment to work as “the degree to which a person’s work performance influences their self-esteem” [55]. Factors influencing affective commitment also include specific personality traits, work environment and conditions (challenges related to tasks, the use of various skills, participation in the decision-making process, organizational fairness, company policy towards employees, manner of communication) or work experience (sense of comfort, security, a sense of the importance of competences). Research shows that leadership style also has a huge impact on employee commitment [56–58]. Leadership style is an administrative tool that, used properly, can positively influence relations with employees, strengthen the hierarchical atmosphere and improve the realization of benefits [59]. The leadership style is a strategy that a leader uses to influence the behavior of subordinates [60]. There are several leadership styles, such as autocratic, bureaucratic, laissez-faire, charismatic, democratic, paternalistic, situational, transactional, and transformational.

However, the variety of environmental contexts, the specificity of tasks performed by subordinates and even differences in research methodology make it difficult to generalize and draw coherent conclusions about the effectiveness of individual management styles. Quite early in research on the motivational effectiveness of leaders, it was noted that there is no single, correct style for managing employee motivation to increase level of commitment.

### Benevolent leadership

The global activity of enterprises, technological progress, highly intense competition and the resulting uncontrolled corporate greed have led employees to expect slightly different behaviors from their leaders. The crisis of confidence in leadership [61] and the uncertainty [62] that many employees have come to operate under have resulted in traditional models and styles of leadership needing to be rethought and rebuilt to more satisfactorily respond to current challenges. An interdisciplinary approach requires that leaders employ models derived from, for example, positive psychology, business ethics, workplace spirituality, appreciative inquiry and corporate social responsibility [16]. A solution has been proposed by Karakas [13] that creates a conceptual model of benevolent leadership based on four thematic streams–morality, spirituality, vitality and community.

Benevolence has featured in the literature on leadership with authenticity, common good and virtuousness as its major components [14, 63]. The concept of benevolent leadership considers leadership to be a process of creating a virtuous cycle of encouraging and initiating positive change in organizations through ethical decision making, creating a sense of meaning, inspiring hope and fostering courage for positive action, and leaving a positive impact for the larger community [16]. Benevolent leaders are those who create visible benefits, actions or outcomes for the common good. The common good in this sense is the benefit of all or most of the members of a community [64, 65]. Benevolent leaders exemplify honest and genuine action at work to the benefit of those around them. Benevolence is defined as the philosophical faith in human goodness and the corresponding faith that people are obligated to use their natural instincts and the developmental attitudes of love and mercy–that they have a tendency towards doing good, kindness and charitable behavior [14]. Benevolent leadership integrates the four streams of the common good–morality, spirituality, vitality and community. Each stream has a purpose and contributes differently to the practice of leaders. All these streams, however, interact and create a comprehensive set of principles for creating a common good in the organization.

The morality aspect derives from business ethics and relates to leaders’ ethical decision-making [3, 66]. The concept of “ethical sensitivity” refers to a leader’s moral reflection and her reflection on what is good and what is bad within the organization [14].

The spirituality aspect refers to understanding employees’ spiritual needs and search for meaning. Spirituality provides leaders with a sense of purpose, meaning at work and connection [4, 67, 68]. The spirituality aspect is related to the leader’s energy, open-mindedness and positive influence on subordinates because, as Kernochan et al. point out, spirituality also involves a deep concern for others. The third stream in the benevolent leadership model is vitality [69]. The authors of the model base this aspect on the concept of appreciative inquiry [70] and positive organizational scholarship [71]. They also refer to positive psychology [72] and positive organizational behavior [73]. All these concepts focus on how a leader can create positive changes in her organizations, especially changes that enhance employee potential. The vitality aspect thus relates to overcoming resistance to change, helping others to develop and unleashing creativity. When subordinates notice the positive commitment of leaders, they try to follow them and show a greater desire to help others.

The last stream in the model is community, which is based on the concept of CSR [74] and corporate citizenship [75]. The essence here is that benevolent leaders fulfill their social responsibilities. Their role is to be open to social problems in the environment, education and ecology, or simply to expand on the idea of sustainable development. The leader’s participation in solving social problems and contributing to societal improvement through organizational innovations is referred to as “community responsiveness” [74, 76]. The “common good” aspect of a benevolent leader must extend beyond the boundaries of the organization.

The most important behavioral manifestations of a benevolent leader include: responsibility and justice; respect and protection of the rights of employees, consumers and employers; making decisions based on ethical guidelines; acting honestly; being aware of one’s own values; observing rules and laws; and promoting moral values at work. A benevolent leader considers the long-term consequences of his own actions. He is aware that his morals affect others. He focuses on doing what is right as opposed to doing what is popular. He observes not only himself but also others in terms of ethical behavior and decision making. He takes into account the problems faced by ethical decision makers. The behavior of a benevolent leader manifests itself in searching for deeper meaning at work, in being more aware of one’s own influence on others. A benevolent leader is compassionate, supportive and helpful. He sets clear goals aimed at the entire community of the organization. He is an idealist who wants to serve humanity. He inspires subordinates to change. He is involved in social innovation and develops shared visions. He inspires hope and courage in his followers. He wants to be a role model. A kind leader believes that his work can positively influence the wider community, uses appropriate tools to solve social problems, builds positive relationships with stakeholders towards whom he is empathetic. He designs and tries to implement solutions to problems such as sustainable development, poverty and education [77].

To conclude, benevolent leadership is thus intended to be the integrity of ethical and spiritual leadership on the one hand and transformational and servant leadership on the other. The effect of his actions is to be trust, stress reduction among employees, openness to change, and the innovation or sustainable development of the company. Benevolent leadership means a holistic approach to business management. The model of benevolent leadership in practice is supposed to mean, among other things, the creation of a common mission, a common sense of purpose, high-quality cooperation and a positive organizational culture. These corporate dimensions are intended to support the creation of positive change in organizations.

### Benevolent leadership and affective commitment

The phenomenon of affective commitment is seen as a special, mutual relationship between the employee and the company, with mutually beneficial outcomes. Committed employees undertake specific actions that help the company to prosper. Meanwhile, a sense of commitment also has positive outcomes for the employee. Many scholars point to the positive impact that affective commitment has on other attitudes. For example, commitment affects employee motivation and performance [78]. Bateman and Strasser evidenced that commitment affects employees’ loyalty and desire to stay in the organization [79]. Affective Commitment is also strongly correlated with Organizational Citizenship Behavior [80–83].

Affective commitment undoubtedly has many positive outcomes for the organization itself [23, 84], and managers should therefore do everything possible to shape and strengthen these attitudes in subordinates. Commitment has been recognized to be a consequence of various leadership styles, such as ethical leadership [85], charismatic leadership [86], transformational leadership [87], active and passive leadership styles [88] and leadership practices [89].

However, there is still limited research into the relationship between benevolent leadership and organizational commitment. The framework of my study is proposed on the basis of Leader Member Exchange theory (LMX). This theory is defined as the interpersonal relationship between a leader and a subordinate that relates to the followers’ outcomes. When the leader and the members are in high-quality relationship, the leader becomes a resource that provides employees with support. This enhances employees’ commitment and makes them psychologically safe [90]. Employees in a high-quality LMX tend to have higher quality exchange relationships with co-workers in the form of social support. It is the benevolent leader who provides social support. Yukl suggests that high-quality exchange relations can result in greater commitment of employees not only in the implementation of tasks, but also in helping the leader achieve goals [8]. In addition, Ansari et al. add that the relationship between leaders and followers is becoming increasibgly important for organizations to learn to build more trust with each other in order to gain greater commitment from subordinates [91].

Alongside LMX theory, Social Exchange Theory (SET) provides a framework in the study of employee–organization relationships. Social exchange is considered an interdependent relationship between two parties because it is a two-way transaction. Social exchanges form the basis of high-quality relationships between employees and their leaders, as well as between employees and their organizations [92] This exchange, therefore, arises when the interactions between the two parties lead to the emergence of a sense of obligation to reciprocate. If reciprocity does not exist, social interaction will end [93].

In other words, social exchange begins when one side takes the initiative to show benevolence and offer benefits and the other side reciprocates with other advantages (e.g. trust, reduced risk of opportunistic behavior, “externalities” that provide economic value and increase efficiency) [94]. Therefore, it can be assumed that benevolent leaders who strive for positive changes in their actions will initiate the process of social exchange. Their concern for the common good will be an initial favor shown to their subordinates, who, in turn, will show positive attitudes and behavior when they want to repay them. One such attitude is organizational commitment, which, according to previous empirical reports, has a significant impact on the process of social exchange [95].

Brown and Treviño argue that the relationship between leader and follower is based on social exchange because it is based on mutual feelings and credibility, not on economics [10]. Benevolent leaders are also ethical leaders, and this means that they possess moral qualities such as honesty and credibility. Therefore, their subordinates are loyal to them and try to achieve the expected goals and show commitment to the organization [96].

Blau [97] argued that the SET could be used to explain the influence of leadership on human interactions. In addition, Hollander and Offermann [98] confirmed the importance of social exchange between leaders and subordinates through their mutual influence and interpersonal perception. In other words, the ability of individuals to behave in a manner consistent with their identity and to refer to others’ identity may be influenced by the social context. It is believed that superiors, through their loyal and moral behavior, can contribute to the development of the same competences and commitment among their subordinates. Erben and Güneşer [21] found that benevolent behavior by leaders encourages commitment. According to social exchange theory, the morality of leaders should lead to the reciprocity of workers. Benevolent behavior by leaders inspires employees and creates an emotional bond between leaders and subordinates. This emotional relationship discourages employees from leaving the company. An employee who displays affective commitment strongly identifies with the goals of the organization and wants to be part of it. Working with a benevolent leader who helps not only subordinates but also the world around him can stimulate a desire to become more committed to the organization. Managers’ care for the common good may encourage employees to identify more closely with the organization. Therefore, I hypothesize that:

**H1**: **There is a positive relationship between benevolent leadership and the affective commitment of employees.**

I intend to verify the first hypothesis (H1) based on the comprehensive BLS scale (Benevolent Leadership Scale)

Benevolent leadership, as mentioned above, is based on four streams: morality, spirituality, vitality and community. It can achieve organizational and social transformation by exploiting the intrinsic motivation of group members [16]. Karakas and Sarigollu combined the morality of leaders with benevolent leadership through “ethical sensitivity”, treating it as a product of virtue, values, ethics, trust and honesty. Ethical sensitivity as the sum of these attributes is a great motivation to integrate employees and therefore also to gain their emotional attachment [73].

The authors of the construct of benevolent leadership have treated the streams of spirituality and vitality as “spiritual wisdom” and “positive engagement”, respectively [14]. Spiritual wisdom combines the leader’s self-awareness, compassion, inspiration, wisdom and reflection. Positive engagement, on the other hand, includes such elements as hope, passion and energy for change. The strengths of both dimensions may be related to the benevolent leader realizing the importance and value of work. Benevolent leadership ensures a high degree of organizational ownership of employees through the perspective of broad development [16]. Hence, there is a possible relationship between the dimensions of “spirtual wisdom” and “positive engagement” of benevolent leadership with the variable of affective commitment.

The last stream, community, was included in Karakas and Sarigollu’s model of benevolent leadership as “community responsiveness.” It indicates the leader’s inclination to act in the fields of CSR, sustainable development or service in helping others [14].

Benevolent behavior in this dimension by the leader may induce an internal motivation of employees to perceive the social context of their work. They will feel that they too can make changes in society. Infecting employees with social responsibility makes them feel united and focused on the same goals, which ultimately leads to identification with the organization through affective commitment.

Until now, studies on the relationship between different dimensions of BL and affective commitment have been limited [14, 99]. The cultural conditions of many countries may be of great importance in the study of these relationships. It is therefore worth getting a picture of the relationship between the various dimensions of BL and emotional attachment. It should also be emphasized again that this research has a slightly different direction–each dimension of benevolent leadership was assessed by employees, and not by the leaders themselves. Therefore, it is worth checking which of these dimensions, in the opinion of employees, is most associated with their emotional attachment. Hypothesis H2 is therefore composed of four parts:

H2a: The morality stream is positively correlated with AC.
H2b: The spirituality stream is positively correlated with AC.
H2c: The vitality stream is positively correlated with AC.
H2d: The community stream is positively correlated with AC.

## Material and methods

### Procedures

Finding benevolent leaders on whom to carry out a study is problematic as, so far, there are no rankings of benevolent leaders. So far, any research in the field of benevolent leadership has been conducted among managerial staff [14, 16]. Leaders made their own judgments about their level of benevolence. This article presents a different view of the leader’s benevolence. According to the author’s proposal, a significant evaluation of the leader’s benevolence can be obtained from the employees themselves. It should be emphasized once again that the author consulted the proposal of such an approach to the BL study with the creator of the BLS (Benevolent Leadership Scale), Fahri Karakas, who decided that it was an interesting direction in the research of benevolent leadership. Thus, in order to investigate the relationship between benevolent leadership and the affective commitment of employees in such a way, the survey was targeted at over 2,000 employees who might “potentially” have a benevolent leader. The survey was sent to employees of enterprises that were included in the list of Polish socially responsible companies (XIII Ranking of Responsible Companies 2019). Directing the survey to the employees of these companies gave a greater chance of reaching companies managed by benevolent leaders. However, the survey was conducted in enterprises diversified in terms of industries, locations and employment. A total of 720 people agreed to participate in the study. After analyzing the results, 305 questionnaires were rejected because the answers clearly confirmed that their supervisor was not a benevolent leader (he showed benevolence only in the ethical dimension). Ultimately, 415 respondents who were subordinates of a benevolent leader took part in the study. Participants were informed that their participation in the study was entirely voluntary. There was no remuneration for participating in this study. The responses of the respondents were anonymous. Most of the surveys were completed online by sending respondents an email containing a link to the survey website. Respondents entered their answers directly online.

The research was approved by the Scientific Research Ethics Committee of the Nicolaus Copernicus University (Permit No. 19/2021/FT). It should be noted that the study does not fall within the field of clinical psychology. Before the research, I obtained written consent from the management of each company. An invitation appeared over the study in which participants were informed about the purpose of the study. Respondents were previously informed that the survey was only about their beliefs about themselves. Before starting the study, each respondent consciously consented to participate. They could withdraw at any time. The study was conducted in accordance with the Declaration of Helsinki.

### Sample

The sample of respondents comprised 415 people employed in Polish companies. Women constituted 54% of respondents. The largest age group (40%) consists of respondents in the 36–45 age range, while 25% are aged 45–55, 17% are 55+ and 18% are under 36. Over 70% of respondents have a higher education plus over five years’ professional experience. It should also be noted that those asked to participate in the study worked in various companies. Thirty-one percent of the respondents worked in production companies, 34% in trading companies, and 35% in services. Thirty-four percent of the respondents worked in small companies (with 10–49 employees), 43% in medium-sized companies (50 to 249 employees) and 23% in large companies (250 or more employees).

The company sample reflects a mix of sectors. There were 12 production, 18 retail and 27 services companies. The survey was conducted in 26 small, 17 medium and 14 large companies.

### Measures

The Meyer and Allen scale was used to measure affective commitment [41]. The scale related to Affective Commitment contains formulations related to the employee’s emotions and feelings towards the organization. The author used a Polish version of the Meyer and Allen scale developed by Bańka, Wołoska and Bazińska [100]. Respondents expressed their attitude towards six statements on a scale of 1 to 5, from 1 meaning “totally disagree” up to 5 meaning “totally agree”. Cronbach’s α coefficient was found to be 0.878 (for the second model the value was 0.817; to ensure good quality of the model, one variable was omitted).

Benevolent leadership was measured using the BLS (Benevolent Leadership Scale) tool proposed by Karakas and Sarigollu [14]. This scale is composed of four dimensions: Ethical Sensitivity, Spiritual Depth, Positive Engagement and Community Responsiveness. Reliability for all dimensions has been found to be acceptable by Karakas [13] (Ethical sensitivity, α = 0.95; spiritual depth, α = 0.84; positive engagement, α = 0.89; community responsiveness, α = 0.81).

Each subscale consists of ten elements defining a specific dimension of benevolent leadership. Therefore, in total, the respondents gave 40 responses on a 5-point scale, where 1 indicated “I definitely disagree”, and 5 “I definitely agree”. It should be emphasized, however, that the present author’s study differed from the studies of the authors of the BLS scale. Karakas and Sarigollou asked managers themselves to answer the questions, and to assess their level on each element of the scale. In this study, however, answers were provided by subordinates, who assessed the level of benevolence of their line manager. So, instead of statements like “I feel and behave like a responsible leader,” phrasings equivalent to “My supervisor behaves like a responsible leader” were used. After obtaining the approval of the authors to adapt the scale, it was translated from English into Polish by two independent experts. The agreed Polish version was back-translated into English by another expert with a satisfactory degree of convergence with the original. Additionally, in this research, all internal subscales yielded an internal reliability alpha greater than 0.70 (ethical sensitivity, α = 0.95; spiritual depth, α = 0.94; positive engagement, α = 0.95; community responsiveness, α = 0.93).

#### Data analysis

The current study used SPSS and AMOS for data analysis purposes. The correlations and reliability were tested using SPSS, whereas confirmatory factor analysis and hypothesis testing were conducted with AMOS.

To test for homogeneity and internal consistency Cronbach’s alpha statistic and convergent validity were calculated. Discriminant validity was checked by confirmatory factor analysis. The hypotheses were tested by structural equation modelling (SEM), which allows the researcher to describe unobservable latent variables. The model was estimated in the SPSS Amos 16 package using the maximum likelihood method. The adopted level of significance was 0.05. Additionally, results were confirmed by bootstrap analysis with the 5,000 samples.

## Results

The relationship between benevolent leadership and affective commitment was investigated using structural equation models (SEMs). The values of Cronbach’s alpha statistics for all analyzed factors were significantly above 0.7, which confirms the good reliability of the scale used to measure them. Additionally, the convergent validity of the variables was evaluated by examining the factor loadings (Table 1).

**Table 1:** Descriptive statistics.

Factor	AVE	Discriminant validity	
		1.	2.
1. Benevolent leadership	0.796	**0.633**	
2. Affective commitment	0.543	0.281	**0.295**

**Notes**: bold values show discriminant validity; p<0.05.

The values of AVE (average variance extracted) are ranged from 0.54 to 0.79. As per the recommendations of Hair, factor loadings above 0.5 are considered significant; thus, the loadings provided a significant contribution for each construct [101]. Diagonal values demonstrate the discriminant validity. These values are higher than the inter-correlations of the variables [102].

The CFA factor analysis is an integral part of the SEM model. It is recommended to perform complete CFA as recommended by Qing et al. by comparing alternative models with the hypothesized model to test the discriminant validity and the possibility of CMB using SEM [103] (Table 2).

**Table 2:** Model fit measures.

Models	Χ^2^	df	Χ^2^/df	delta Χ^2^	IFI	TLI	CFI	RMSEA
Baseline model	364.582	76	4.797		0.928	0.913	0.928	0.087
1-Factor model	990.336	77	12.862	625.754	0.772	0.730	0.771	0.172

**Abbreviations:** χ^2^, Chi square; df, Degree of Freedom; IFI, Incremental Fit Measures; CFI, Comparative Fit Index; RMSEA, Root Mean Square Error of Approximation.

Table 2 confirmed the findings of CFA, where the baseline model is compared with a one-factor model. As evidence in Table 2, the baseline model of our study provides the most appropriate fit indices.

**Fig 1:**
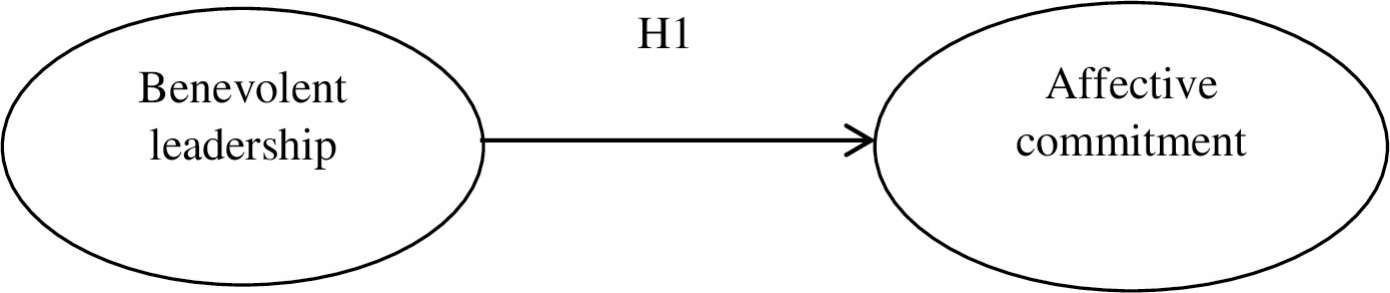
SEM 1: Verification of relationship between benevolent leadership and the affective commitment of employees.

The identified factors allowed SEM model to be constructed. This model was designed to verify the first hypothesis that benevolent leadership is positively correlated with the affective commitment of employees (see Fig 1).

The model was estimated in the SPSS Amos 16 package using the maximum likelihood method. The adopted level of significance was 0.05. Table 3 contains the parameters for the estimated external model (factor analysis), and Table 4 for the internal model.

**Table 3:** SEM 1 external model assessment results.

Dependence	Parameter	Assessment of parameter	P value
P2 ← Benevolent leadership	α_1_	0.837	
P4 ← Benevolent leadership	α_2_	0.754	0.000
P9 ← Benevolent leadership	α_3_	0.812	0.000
P16 ← Benevolent leadership	α_4_	0.770	0.000
P19 ← Benevolent leadership	α_5_	0.823	0.000
P22 ← Benevolent leadership	α_6_	0.843	0.000
P26 ← Benevolent leadership	α_7_	0.857	0.000
P28 ← Benevolent leadership	α_8_	0.867	0.000
P41 ← Affective commitment	α9	0.620	0.000
P42 ← Affective commitment	α10	0.752	
P43 ← Affective commitment	α11	0.779	0.000
P44 ← Affective commitment	α12	0.789	0.000
P45 ← Affective commitment	α13	0.794	0.000
P46 ← Affective commitment	α14	0.705	0.000

**Table 4:** SEM 1 internal model assessment results.

Dependence	Parametr	Assessment of parameter	Assessment of standardized parameters	P value
Benevolent leadership → Affective commitment	β1	0.466	0.601	**0.000**

**Notes**: bold values show discriminant validity; p<0.05.

All factor loadings are greater than 0.6 and p values for all of them are less than 0.05 (Table 3). Thus, they are statistically significant. Benevolent leadership is positively related to affective commitment of employees (β1 = 0.601; p = 0.000). The more benevolent leadership qualities a manager has, the more affective commitment employees show. Thus, the first hypothesis was confirmed. The values of the measures of the degree of model fit are as follows: CFI = 0.928; PNFI = 0.660, RMSEA = 0.087. While the CFI value undoubtedly indicates a high-quality model, the RMSEA is slightly too high, but it still confirms a correct fit of the model to the data.

Due to the relatively high RMSEA in model 1, the results were subjected to further verification. To this end, a bootstrap procedure was used to re-estimate the model parameters using a maximum likelihood estimator. The procedure was carried out on the basis of 5,000 samples and allowed parameter biases and their standard errors to be calculated, and load-adjusted confidence intervals to be determined at the 95% level. The results are summarized in Table 5.

**Table 5:** Estimation results of SEM 1 model by bootstrap procedure.

Parameter	Estimate	Bias	S.E. Bias	Lower	Upper	P value
**SEM 1 Model (relationship with affective commitment)**						
α1	0.837	-0.001	0.000	0.790	0.873	0.001
α2	0.754	0.000	0.000	0.693	0.804	0.000
α3	0.812	0.000	0.000	0.763	0.842	0.001
α4	0.770	0.000	0.000	0.714	0.820	0.000
α5	0.823	0.000	0.000	0.778	0.863	0.001
α6	0.843	0.000	0.000	0.791	0.871	0.001
α7	0.857	0.000	0.000	0.801	0.889	0.001
α8	0.867	0.000	0.000	0.821	0.902	0.001
α9	0.620	-0.001	0.001	0.537	0.698	0.000
α10	0.752	-0.001	0.001	0.681	0.822	0.000
α11	0.779	0.000	0.000	0.717	0.833	0.001
α12	0.789	0.000	0.000	0.712	0.838	0.001
α13	0.794	-0.001	0.000	0.723	0.842	0.000
α14	0.705	0.000	0.001	0.641	0.781	0.001
β1	0.601	-0.003	0.001	0.505	0.691	0.000

The results show that most of the factor loadings of the benevolent leadership variable (α1–α8) had no statistical bias loading at all. It is assumed that the parameter bias value is statistically insignificant when the absolute value of the bias’s standard error is greater than that of the bias itself. However, the existence of bias in the model parameters does not determine the parameters’ statistical significance. The bias-adjusted confidence intervals confirm the significance of all parameters estimated by the maximum likelihood method, i.e. the estimated values are within their ranges and none of the ranges contain a value of 0.

In order to look at the relationship between particular benevolent leadership dimensions and affective commitment, a second structural equation model, SEM 2, was constructed. Observable variables characterizing individual factors were selected based on substantive content, ensuring the highest possible factor loadings in the confirmatory factor analysis, suitable values of Cronbach’s alpha reliability statistics, and sufficient SEM model fit to the data.

The values of the Cronbach’s alpha statistics for all analyzed factors were significantly above 0.7, which confirms the good reliability of the scale used to measure them. Additionally, the convergent validity of the variables was evaluated by examining the factor loadings (Table 6).

**Table 6:** Descriptive statistics.

Factor	AVE	Discriminant validity				
		1.	2.	3.	4.	5.
1. Social responsibility	0.753	**0.567**				
2. Spirituality	0.752	0.326	**0.564**			
3. Morality	0.845	0.273	0.472	**0.713**		
4. Vitality	0.829	0.395	0.338	0.632	**0.687**	
5. Affective commitment	0.557	0.467	0.468	0.226	0.241	**0.311**

**Notes**: bold values show discriminant validity; p<0.05.

The values of AVE are ranged from 0.56 to 0.85. These values revealed the convergent validity because these are greater than 0.50 [101]. Diagonal values demonstrate the discriminant validity. These values are higher than the inter-correlations of the variables [102].

According to the recommendation of Qing et al., also compared here are alternative models with the hypothesized model to test the discriminant validity and the possibility of CMB using SEM [103] (Table 7).

**Table 7:** Model fit measures.

Models	Χ^2^	df	Χ^2^/df	delta Χ^2^	IFI	TLI	CFI	RMSEA
Baseline model	597.617	160	3.735		0.941	0.922	0.941	0.081
4-Factor model (Social resp. + Spirituality)	820.57	164	5.003	222.953	0.912	0.886	0.911	0.098
4-Factor model (Social resp. + Morality)	1081.025	164	6.592	483.408	0.877	0.841	0.876	0.116
4-Factor model (Social resp. + Vitality)	711.485	164	4.338	113.868	0.926	0.905	0.926	0.090
4-Factor model (Spirituality + Morality)	793.343	164	4.837	195.726	0.915	0.871	0.915	0.096
4-Factor model (Spirituality + Vitality)	875.26	164	5.337	277.643	0.904	0.877	0.904	0.102
4-Factor model (Morality + Vitality)	1227.742	164	7.486	630.125	0.857	0.816	0.856	0.125
3-Factor model (Social resp. + Spirituality + Morality)	1193.12	167	7.144	595.503	0.862	0.825	0.861	0.122
3-Factor model (Social resp. + Morality + Vitality)	973.127	167	5.827	375.51	0.891	0.863	0.891	0.108
3-Factor model (Social resp. + Spirituality + Vitality)	1405.714	167	8.417	808.097	0.833	0.789	0.832	0.134
3-Factor model (Spirituality + Morality + Vitality)	1339.559	167	8.021	741.942	0.842	0.800	0.841	0.130
2-Factor model (Social resp. + Spirituality + Morality + Vitality)	1516.133	169	8.971	918.516	0.819	0.773	0.818	0.139
1-Factor model	1800.907	170	10.594	1203.29	0.780	0.727	0.779	0.152

**Abbreviations**: χ2, Chi square; df, Degree of Freedom; IFI, Incremental Fit Measures; CFI, Comparative Fit Index; RMSEA, Root Mean Square Error of Approximation.

**Fig 2:**
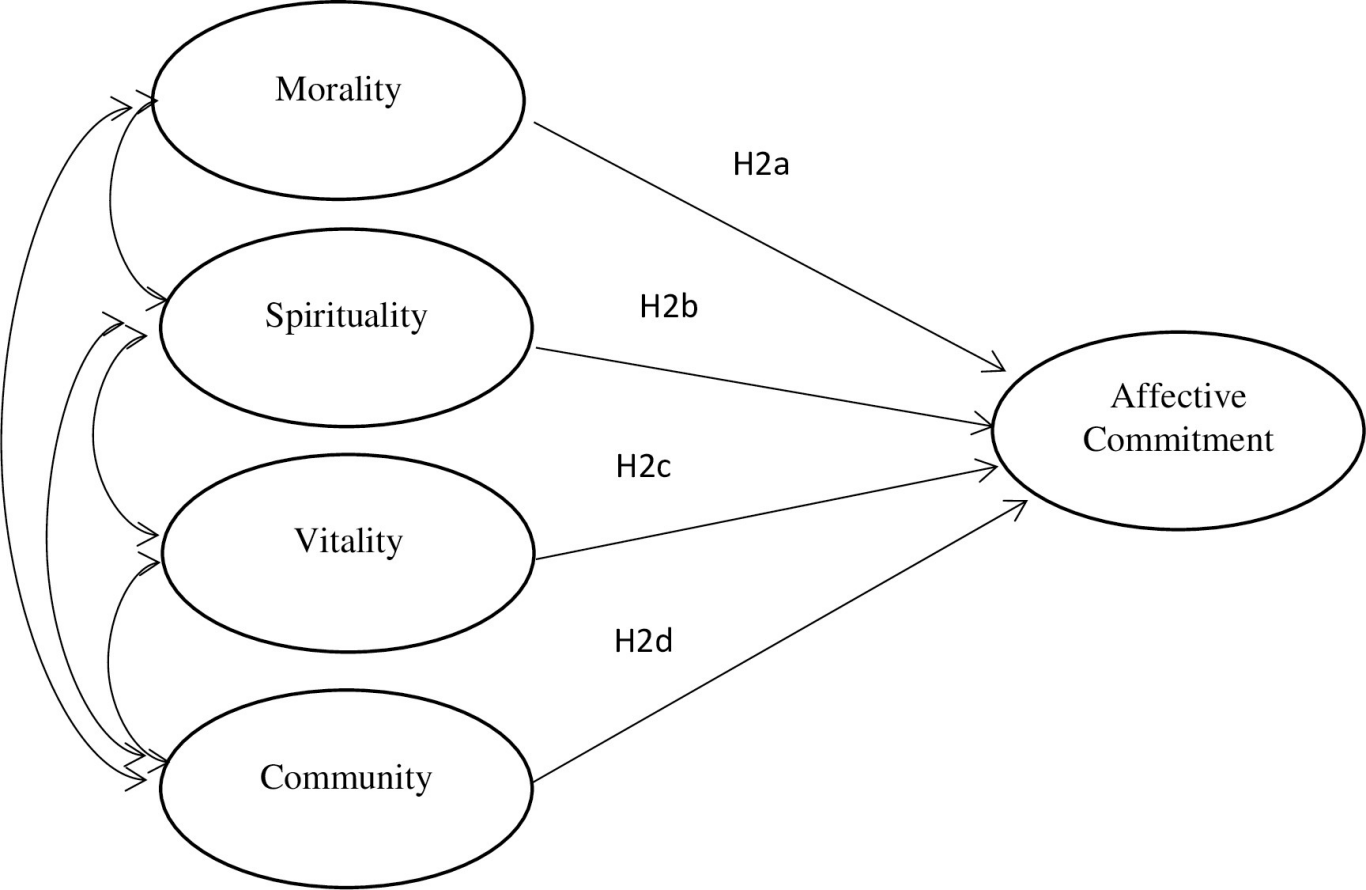
SEM 2: Verification of the relationships between individual dimensions of benevolent leadership and employee affective commitment.

Table 7 confirmed the findings of CFA, where the baseline model is compared against twelve other models (four-factor, three-factor, two-factor, and one-factor). As evidence from Table 7, the baseline model of our study provides the most appropriate fit indices. The identified factors allowed an SEM model to be built to verify the second hypothesis (H2a, b, c, d). The estimated model is presented in Fig 2.

The model assumes that there are connections between the various dimensions of benevolent leadership. Because it was impossible to substantively determine the cause–effect relationships for these dimensions, only the correlations between them were included in the model.

Model 2 was also estimated using the maximum likelihood method in the SPSS Amos 16 package. The adopted level of significance was 0.05. Table 8 contains the parameters for the estimated external model (factor analysis), and Table 9 for the internal model.

**Table 8:** SEM 2 external model assessment results.

Dependence	Parameter	Assessment of parameter	P value
P2 ← Social responsibility	α_1_	0.850	0.000
P3 ← Social responsibility	α_2_	0.840	
P4 ← Social responsibility	α_3_	0.854	0.000
P7 ← Social responsibility	α_4_	0.626	0.000
P9 ← Spirituality	α_5_	0.813	
P16 ← Spirituality	α_6_	0.860	0.000
P17 ← Spirituality	α_7_	0.828	0.000
P18 ← Spirituality	α_8_	0.688	0.000
P19 ← Morality	α_9_	0.884	
P20 ← Morality	α_10_	0.950	0.000
P21 ← Morality	α_11_	0.933	0.000
P22 ← Morality	α_12_	0.911	0.000
P26 ← Vitality	α_13_	0.892	
P28 ← Vitality	α_14_	0.915	0.000
P29 ← Vitality	α_15_	0.883	0.000
P30 ← Vitality	α_16_	0.861	0.000
P41 ← Affective commitment	α_17_	0.642	
P42 ← Affective commitment	α_18_	0.695	0.000
P44 ← Affective commitment	α_19_	0.768	0.000
P45 ← Affective commitment	α_20_	0.805	0.000

**Table 9:** SEM 2 internal model assessment results.

Dependence	Parameter	Assessment of parameter	Assessment of standardized parameters	P value
Social responsibility → Affective commitment	β1	0.311	0.425	**0.002**
Spirituality → Affective commitment	β2	0.239	0.280	**0.019**
Morality → Affective commitment	β3	0.241	0.055	**0.031**
Vitality → Affective commitment	β4	0.315	0.034	**0.042**

**Notes**: bold values show discriminant validity; p<0.05.

The results for the external model (see Table 8) indicate that all factor loadings are statistically significant. When interpreting the results, it should be noted that all components of benevolent leadership had a positive relationship with affective commitment (Table 9). However, the greatest was found in the community dimension.

Additionally, Table 10 presents the values of the correlation and covariance coefficients between the components of benevolent leadership.

**Table 10:** Values of correlation and covariance coefficients between leadership dimensions.

Dependence	Parameter	Correlation coefficient	Covariance	P value
Social responsibility ↔ spirituality	Π1	0.791	0.793	0.000
Social responsibility ↔ morality	Π2	0.757	0.863	0.000
Social responsibility ↔ vitality	Π3	0.892	0.952	0.000
spirituality ↔ morality	Π4	0.850	0.832	0.000
spirituality ↔ vitality	Π5	0.799	0.808	0.000
morality ↔ vitality	Π6	0.795	0.914	0.000

**Note**: p<0.05.

Analyzing the results in Table 10, conclusions can be drawn regarding individual dimensions of benevolent leadership. Correlation in each pair on social responsibility, spirality, morality and vitality is between 0.750 and 0.900. All analyzed dimensions correlate positively with each other, so there is a high probability that if a leader displays one BL dimension, he will also display another. When assessing the degree of model fit to the empirical data it should be noted that CFI = 0.941, PNFI = 0.702 and RMSEA = 0.079, which allows us to conclude that the model had a correct and acceptable fit to the empirical data.

**Fig 3:**
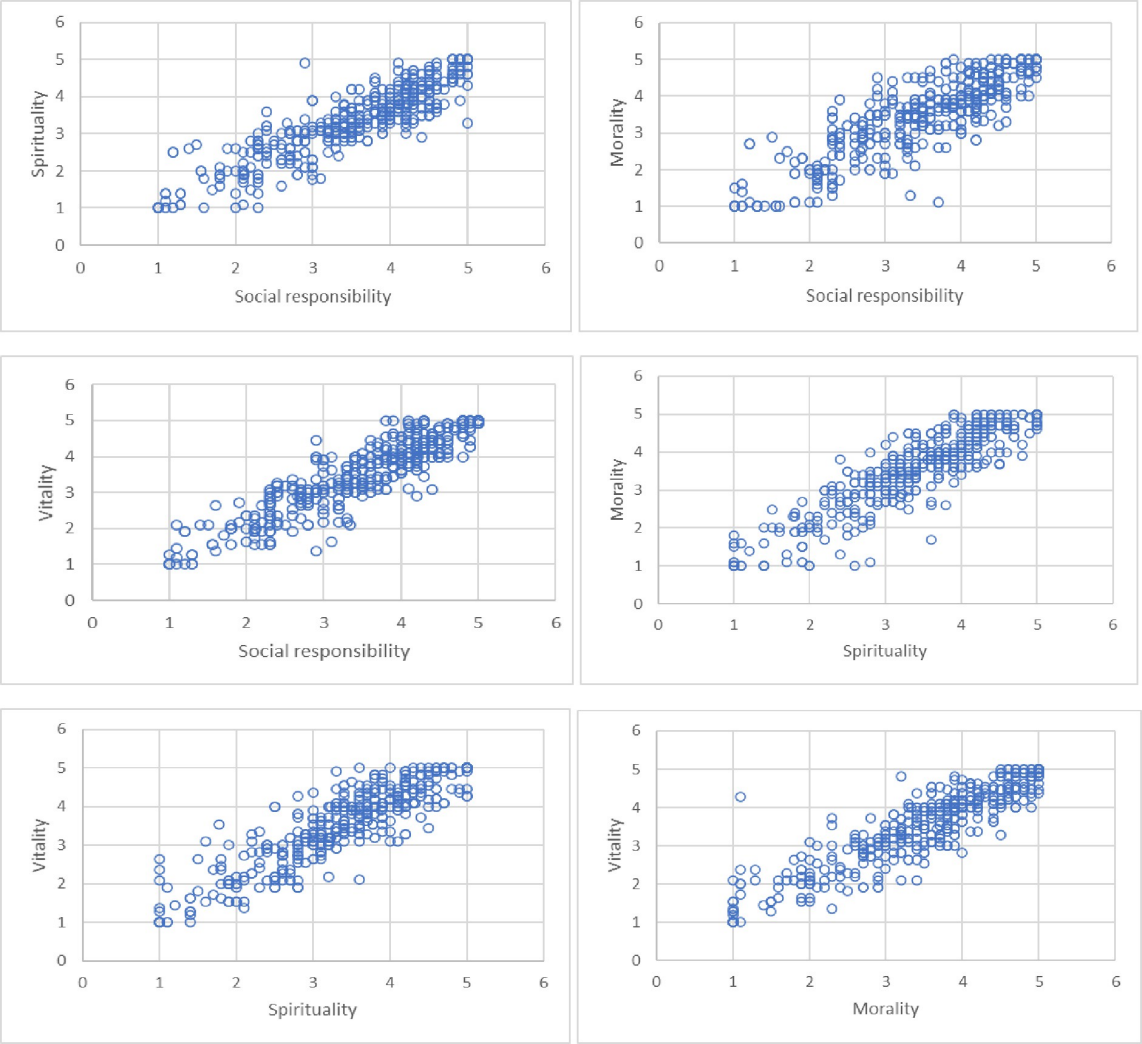
Interdependencies between four dimensions of benevolent leadership.

**Fig 4:**
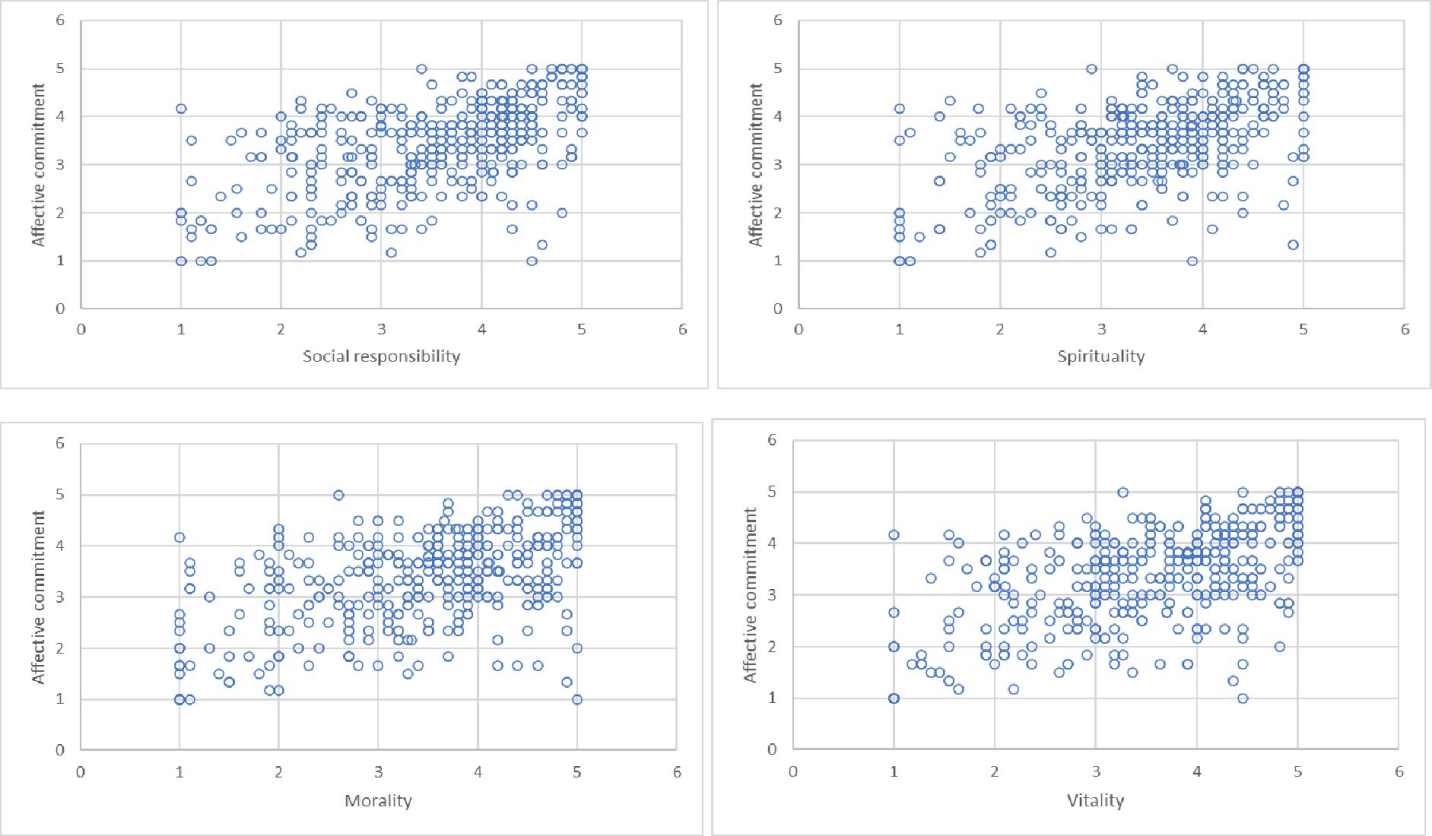
Dependencies between four dimensions of benevolent leadership and affective commitment.

The relationships between the dimensions of benevolent leadership and affective attachment, confirmed by the SEM model, are also visible in the individual figures (see Figs 3 and 4).

## Discussion

Prior research suggests that there is the strong positive relationship between leadership and affective commitment [104–108]. Leadership is an organizational factor considered to be a key determinant of organizational commitment [109]. Many scholars have noted that leadership style correlates with employee commitment [110–112]. Employees’ commitment and identification with the organization increase if they trust their leader and if these leaders satisfy the needs of their subordinates and support them [113]. This makes the concept of benevolent leadership, which is based on the pursuit of the common good, all the more important. Luu clearly emphasizes that BL leads to many positive attitudes and behaviors among employees [114].

The aim of this study was primarily to better understand the relationship between benevolent leadership and affective commitment. The assessment of this relationship was made in the context of Polish organizations, which had not been previously studied. In addition, the benevolence of the leader was assessed by the employees (subordinates) themselves, and not, as before, by managers. Overall, the results indicated that benevolent leadership greatly influences the emotional attachment of employees. This is in line with previous research results [14, 20, 99].

The results are consistent with social exchange theory [115] and the norm of reciprocity [116]. According to these theories, an individual’s volunteering and commitment are motivated by the belief that it will be reciprocated. As social exchange theorists suggest, workers are likely to “exchange” their commitment for the support of leaders [115, 117, 118]. Cropanzano and Mitchell further argue that leaders attending to the well-being of their subordinates can, within the framework of social exchange theory, foster positive employee attitudes [95]. Thus, employees reciprocate the benevolence of leaders in their attachment to and identification with the organization [14, 117]. Thus, they exhibit the most desirable dimension of commitment–affective commitment. When employees observe benevolent leadership behavior at work, they become more likely to duplicate that leadership behavior so that they can contribute to the common good. Employees identify with the goals of the organization and want to remain part of it. Working with benevolent leaders who support their colleagues and the organization can make employees want to identify with their organization, which strengthens their emotional commitment [99].

The study findings showed a strong positive relationship between benevolent leadership and affective commitment. Thus, the more benevolent the leader, the stronger the employee’s bond with the entire organization. This means identifying not only with the organization itself, but also with the leader in particular, as has been demonstrated by previous studies [19, 119].

The present study has confirmed that, in the Polish context too, morality, spirituality, vitality and community are four streams of benevolent leadership. They all have a positive effect on the affective commitment of employees. The morality aspect relates to leaders’ ethical decision-making [3, 66]. A leader reflecting on what is good and what is bad, and thus having an “ethical sensitivity” [120], inspires the respect and trust of her subordinates. The employee is more engaged and productive because he feels obliged to reciprocate with the benevolent leader [119]. The stream of spirituality is also important, as it relates to the search for meaning. Spiritual benevolent leaders have qualities that cause their subordinates to trust and follow them [15]. Spirituality means a deep concern for subordinates, and this increases the emotional, and therefore affective, bond with the organization. Affective Commitment is also influenced by the vitality stream, which is associated with, among other things, introducing changes in the organization and unleashing creativity. The positive commitment of benevolent leaders makes employees identify with their workplace. The study results indicate that affective commitment is most strongly affected by the last stream–community. This therefore confirms the result of Karakas and Sarigollu [14]. In their research in Canada, the community stream was also the most significant.

According to the theory of social learning [121], a socially responsible leader encourages such values in his followers. Affective commitment is the result of, among other things, recognition for the enterprise. Thus, if an employee witnesses good being done for the wider community, the organization gains in his estimation in the long run, which fosters the building of emotional bonds. However, in A. Naami’s study in Iran, the community stream turned out to have the least impact on affective commitment among the four BL dimensions. The most significant was the stream of morality [99]. Therefore, it can be presumed that cultural conditions may be important in the perception, but also in the showing of benevolence by leaders. It is therefore worth carrying out further analyses in this regard.

### Conclusions and implications

Sustainable economic development requires many organizations to look at their activities with a broader perspective. It is not only profits that count, but also taking care of the common social good. Benevolent leadership is a response of sorts to current challenges. The benevolent leader displays ethical, spiritual and transformational behaviors while also being socially responsible. Her leadership is principled and she is extremely brave. The research results clearly showed that benevolent leadership is positively correlated with affective commitment. Therefore, if the employee is a subordinate of a benevolent leader, he will exhibit similar behavior. In addition, he will be emotionally bonded with the organization, which in turn leads to him identifying with it and staying with it for the long term. As mentioned in the article, the benefits of affective commitment are enormous. Since benevolent leadership stimulates AC, the human resources policy in the organization should be designed to identify, stimulate and support its potential leaders’ pursuit of benevolent behavior. As early as during candidate recruitment and selection, personality tests can be conducted to reveal candidates’ tendency for benevolent leadership. By employing such candidates, the organization will incur fewer costs in training and shaping the desired leadership style.

Benevolent leadership brings positive changes to organizations. It focuses on creating good for the wider community. Therefore, bearing in mind the importance of BL, organizations should also focus on developing benevolence among employees, especially those with potential as future leaders.

The presented study makes a significant contribution to the literature, especially in the field of leadership. First, it confirms that benevolent leadership as a synthetic construct is also of use in other cultural contexts. Secondly, it fills the research gap regarding the effect of benevolent leadership on the affective commitment of employees. To the author’s knowledge, only the creators of the construct have tested the influence of BL on affective commitment [14]. However, the subjects of their research were managers who assessed the level of benevolence themselves. Thirdly, the study findings support predictions derived from social exchange theory. Benevolent leaders, and thus fair and trustworthy leaders, will motivate their subordinates to emulate positive behavior. If treated well, employees will strive to reciprocate, which will ultimately affect the employee’s emotional bond with the organization. Fourthly, the study also relates to leader–member exchange (LMX) theory, which focuses on the dyadic relationship between superior and subordinate. As noted by Zhao et al., according to LMX theory, superiors provide their subordinates with means of exchange, such as trust, respect and care [122]. Subordinates can reciprocate to their superiors through pro-social organizational behavior. The benevolent leader who focuses on pursuing the common good provides subordinates with the aforementioned means of exchange, and subordinates are prone to reciprocate these values in commitment.

Undoubtedly, the study also supports the existence of a psychological contract between leader and subordinate [123]. One factor that results from this contract and that conditions the employee’s commitment is fair remuneration. A benevolent leader rewards employees in just such a manner.

### Limitations and future work

There are of course some limitations to this study. First, despite the fact that the participants were selected from different organizations, from different sectors, and in different regions of Poland, the results cannot be generalized, since the sample was not selected probabilistically. Second, the study looked at the individual level only. It would be worth knowing the impact that benevolent leadership has on, for example, the commitment and performance of an entire team. Third, because this study was cross-sectional, no conclusions can be drawn about the direction of causality in the model. Longitudinal research could indicate, for example, the processes by which benevolent leaders make ethical decisions or take actions for the general good. Fourth, a survey method was adopted in this study. Qualitative research could undoubtedly prove very valuable here. In-depth interviewing methodologies can provide rich descriptions of how benevolent leaders create positive change in organizations.

This survey only included company employees. It would be worthwhile also undertaking research in public institutions, which are by definition established to serve society. Another interesting direction is the issue of the antecedents of BL. So far, attention has been focused only on emotional intelligence, but a leader’s qualities, flexibility and openness are also important. Another potential area of future research is the relationship between benevolent leadership and other organizational outcomes such as satisfaction or creativity. The results of the study also indicated the crucial importance of a different BL dimension than in the Iranian study.

It is therefore suggested the need to conduct broader research in the context of different cultures.

There is still a lot of room for further research on the subject of benevolent leadership. However, this work has broadened the knowledge in this area, indicating that benevolent leadership is positively correlated with the affective commitment of employees.
